# Evaluation of a two-step model of opportunistic genomic screening

**DOI:** 10.1038/s41431-024-01592-0

**Published:** 2024-03-25

**Authors:** Melissa Martyn, Ling Lee, Alli Jan, Elly Lynch, Rona Weerasuriya, Anaita Kanga-Parabia, Clara Gaff

**Affiliations:** 1 Melbourne Genomics Health Alliance, Parkville, VIC 3052 Australia; 2 Department of Paediatrics, University of Melbourne, Parkville, VIC 3052 Australia; 3 Murdoch Children’s Research Institute, The Royal Children’s Hospital, 50 Flemington Road, Parkville, VIC 3052 Australia; 4 Central Coast Local Health District, NSW Health, Gosford, NSW 2250 Australia; 5 Victorian Clinical Genetics Services, The Royal Children’s Hospital, 50 Flemington Road, Parkville, VIC 3052 Australia; 6 Australian Red Cross, 23-47 Villiers Street, North Melbourne, VIC 3051 Australia

**Keywords:** Health policy, Personalized medicine

## Abstract

Increasing use of diagnostic genomic sequencing is pushing health services to confront the issue of opportunistic genomic screening (OGS). To date, OGS has been offered concomitant with diagnostic testing. In contrast, we piloted a service offering OGS after return of diagnostic testing results. Evaluation was designed to provide insights for future models of service and included patient surveys at three time points, semi-structured interviews with genetic counsellors (GCs) and a focus group with medical scientists. Uptake was relatively low: 83 of 200 patients approached (42%) attended the OGS service, with 81 accepting OGS. Whilst many who declined to attend the service cited practical barriers, others gave reasons that indicated this was a considered decision. Despite specific genetic counselling, one third of patients did not understand the scope of re-analysis. Yet after post-test counselling, all respondents with novel pathogenic additional findings (AF) understood the implications and reported relevant follow-up. Recall was high: five months after last contact, 75% recalled being offered OGS without prompting. GC interviews and patient survey responses provide insights into complexities that influence patient support needs, including diagnostic status and AF result type. There was no consensus among patients or professionals about when to offer OGS. There was a clear preference for multiple, flexible methods of information provision; achieving this whilst balancing patient support needs and resource requirements is a challenge requiring further investigation. Decisions about whether, when and how to offer OGS are complex; our study shows the two-step approach warrants further exploration.

## Introduction

The advent of genomic testing for diagnostic purposes has brought with it the possibility of opportunistic genomic screening (OGS) for future disease. That is, a deliberate, pre-emptive analysis of the genome for conditions unrelated to the initial indication for testing [1]. OGS has been contentious, particularly in collectively funded healthcare systems, with extensive ethical debate regarding logistics, cost, medicolegal issues, and psychological impact [2]. The American College of Medical Genetics and Genomics [3] position is that patients should be offered OGS for medically actionable disease-causing gene variants, which we term ‘additional findings’ (AF), unrelated to the initial indication for testing. A more cautious approach has been taken in Europe [4] and Canada [5], where professional organisations stress the necessity of dedicated counselling and informed patient choices. In Australia, clinical laboratories do not routinely offer OGS to those undergoing genomic diagnostic tests [6] and policy makers have identified the need to develop and promote a national, principles-based approach [7]. The European Society of Human Genetics have called for OGS pilot and evaluation studies to support decision-making about OGS [4].

Process evaluation of pilots is essential to inform future implementations of OGS beyond a research context. To date, the majority of reported OGS delivery is the ‘concurrent model’, wherein OGS is offered in the same consultation as diagnostic testing [8–10] or at the time of result delivery [11]. This method of service delivery offers system efficiency benefits, with fewer contacts required with clinicians and integrated analysis and reporting for medical scientists. However, it potentially reduces informed decision-making about OGS—in the context of undergoing genetic analysis for a diagnosis, patients may be more likely to be eager for any information without fully understanding the potential implications of AF [12]. Poor recall of OGS decision-making in this model has been reported [13]. Genetic counsellors (GC) report that returning results from diagnostic testing and OGS at the same time is challenging, particularly when both were significant [14], suggesting this be done in separate appointments. The French Society of Predictive and Personalised Medicine [15] suggests performing OGS for cancer predisposition genes simultaneously with diagnostic testing and allowing patients to either confirm or refuse OGS results at the time of result delivery. A pilot of a similar model [16] resulted in several cases where clinically important medical information was known to health professionals that the patient chose not to know.

The process by which OGS is offered and provided is likely to influence outcomes such as patient uptake, knowledge, decisional conflict and regret, and participation in medical follow-up for pathogenic AF. To address the challenges inherent in providing informed consent concurrently for two different genomic analyses with very distinct consequences, we proposed an alternative approach where OGS is both offered and performed after the primary clinical test has been completed and results returned [17]. To evaluate this two-step model, we established a proof-of-concept service. Adults who had previously received the results of clinical exome sequencing from their clinician were offered OGS on stored data for 27 adult-onset conditions. The service provided pre-test genetic counselling to all those who accepted the invitation to learn about the OGS service and post-test genetic counselling to those who proceeded with OGS. We have previously reported our evaluation of resource implications of different service delivery models, drawing on data from this study [18]. Here we report the evaluation of our proof-of-concept service, using mixed methods to explore key elements of delivery including patient uptake, decision-making, understanding, recall and patient and professional perspective on the service and preferences for future models.

## Methods

The protocol has been described in detail elsewhere [17]. The following summarises key aspects of the clinical procedures and study methodology. We use OGS to refer to the process and AF for the test result.

### Patient participants

Two hundred patients were randomly selected using the approach previously described [17] from a database of patients who received genomic sequencing within clinical care through a Melbourne Genomics Health Alliance funded clinical service design project. Eligibility criteria were: adults over 18 years who had undergone germline exome sequencing for diagnosis of their own clinical condition or as a parent of an affected child (trio testing); consent to be recontacted for further research; English language ability. Potential patient participants received information about OGS and were asked to indicate if they wished to be contacted by a counsellor to learn more. It was emphasised that agreeing to be contacted did NOT oblige them to accept analysis for AF.

### Clinical procedures

In-person genetic counselling appointments were arranged for those who expressed interest in knowing more about the OGS service. The research consent form and a decision support tool [17] (Supplementary File 1) were sent prior to the appointment. Pre-test counselling was conducted in genetic services at metropolitan public hospitals in Victoria, Australia, by experienced GCs trained in OGS [17]. Patients who elected to proceed with OGS signed a clinical consent form for reanalysis of their stored genomic data. Genomic data was then analysed by an accredited clinical laboratory for 58 genes known to cause 27 adult-onset conditions with funded management pathways available in the Victorian public healthcare system (Supplementary File 2). OGS results of were discussed at multidisciplinary meetings. Results were returned to patients by a GC either by phone if the patient had a negative or known result, or in person if there was a pathogenic AF.

### Data collection

Data were collected from patients, GCs and medical laboratory scientists.

#### Patient participant data

Patient demographics, initial testing outcome and hypothetical interest in reanalysis for AF were obtained from the primary study database [17]. Uptake of the OGS service, reasons for decision (including reasons for declining) and OGS decision were recorded in a database specific to this study.

Surveys, either electronic or hard copy according to preference, were administered to patient participants at three time points as previously described [17]: survey 1 was administered to all patient participants after pre-test counselling and focused on decision making and understanding; survey 2 was administered only to those who accepted OGS after return of results and focused on understanding of result and decision regret; survey 3 was a short phone survey administered to all patient participants at least 5 months after last contact with a GC and focused on recall and decision regret. Questions on preferences for future service provision were included in S1 and S2.

#### Professional participant data

Semi-structured interviews were conducted with six of the ten GCs who provided counselling for the OGS service. Interviews focused on their experiences providing OGS to patients, views on workforce support needs and on future delivery of OGS. A focus group was conducted with ten medical laboratory scientists involved in the OGS service exploring the processes used for AF identification and reporting, and perspectives on OGS in the future.

### Data analysis

Survey data were captured and managed using REDCap [19, 20] electronic data capture tools hosted at Murdoch Children’s Research Institute. Descriptive analysis of quantitative data was performed with Stata [21], including participation and response statistics, and univariate analysis of demographic features with a Pearson-Chi squared test. Results with a *p* value less than 0.05 were deemed statistically significant. Qualitative data from GC interviews and the focus group were recorded and transcribed verbatim. Transcripts from GC interviews and the medical scientists focus group and open-ended responses from patient surveys were imported into the qualitative data analysis package NVivo [22]. Transcripts were analysed using an inductive deductive hybrid approach to thematic coding [23]. Deductive coding was used to assign the content of transcripts into pre-determined categories e.g. patient understanding of results; inductive coding was then used to identify emergent concepts within each category. Open-ended survey responses were analysed inductively. All qualitative analyses were synthesised and discussed within the study team.

## Results

**Fig. 1:**
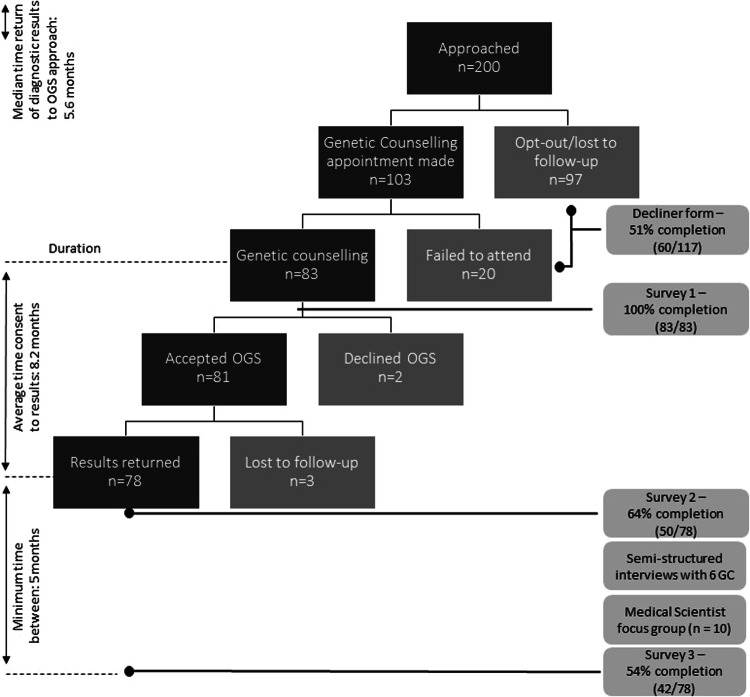
Participation in the opportunistic genomic screening service and evaluation activities.

Participation in the OGS service and evaluation activities is described in Fig. 1. Note that denominators given for survey responses in text vary slightly, due to missing answers.

### Uptake of offer to attend the OGS service

**Fig. 2:**
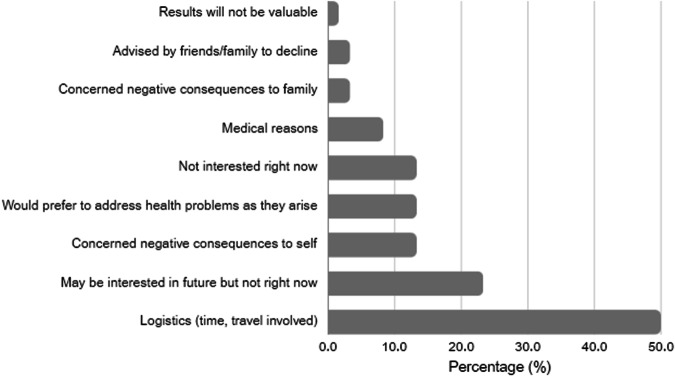
Reasons for declining the opportunity to learn about opportunistic genomic screening.

Patients offered the opportunity to attend the OGS service were well distributed across socio-demographic variables (income and educational levels) (Supplementary File 3). Of the 200 approached, 83 accepted the offer to attend (42% uptake). There were no significant differences in patient characteristics (age, sex, location, country of birth, education, previous diagnosis, time from completing initial testing to being approached) between those who accepted and those who declined the opportunity to attend the OGS service (Supplementary File 3). Eighty per cent of those who attended the OGS service were undiagnosed after diagnostic genomic testing. Of those who did not attend the service, 51% provided reasons (Fig. 2). Practical considerations e.g. travel time were most commonly cited but concerns about the value or impact of OGS or a preference to deal with medical issues as they arise were also nominated. Thirty-one respondents who declined the OGS offer cited only practical considerations that could be overcome with a true open-ended OGS offer (i.e. only ‘logistics’ or ‘not the right time’). Adding this to the 83 who attended the OGS service gives a theoretical maximum uptake of 57%. Four couples tested as part of a trio opted-in to the service of the 12 couples randomised to be approached. The small number of trio participants mean we are unable to explore differences between this group and adults who’d had diagnostic testing themselves.

### Decision-making and uptake of OGS (analysis for AF)

During diagnostic testing, patients were asked about their hypothetical interest in undergoing reanalysis for AF for serious, actionable conditions. Responses available for 146 of the 200 people approached showed 95% (*n* = 138) expressed hypothetical interest. After genetic counselling, 81 of the 83 patients consented to OGS, giving an actual uptake of reanalysis for AF from the same population of 41% (81/200).

The majority of patients (95%) in survey 1 indicated the level of information they received prior to making a decision about OGS was ‘about right’ (*n* = 79/83). Over 90% (73/78) reported the pre-test discussion with the GC was helpful. Open comments indicated patients valued the opportunity to ask questions. One patient commented that, due to dyslexia, they prefer discussions to reading. Two commented that the discussion was a formality, as they had made their decision already and two said a phone consultation would be sufficient. Whilst the amount of information in the decision support materials was considered to be ‘about right’ (67/79; 85%), some (21/78, 27%) perceived it to be biased in favour of OGS. Few respondents (3/82, 4%) had high decisional conflict.

### Pre-test understanding

Patient survey 1 included six questions to assess understanding of OGS. Most respondents correctly understood the implications of a pathogenic AF for themselves and their family. Patients were asked to indicate using a slider how likely a pathogenic AF result was. Less than a quarter nominated a value of 10% or lower, i.e. within the range regarded as ‘correct’. A third of respondents did not understand that OGS was for a select group of conditions with known treatment or intervention: 23 thought analysis would encompass all known genetic conditions and five thought it related to the initial diagnostic testing.

### Return of results—patient and professional perspectives

Of the 81 patients who accepted OGS, six had novel pathogenic AF (Supplementary File 4), all of whom completed survey 2. All survey 2 respondents correctly recalled their AF results. Most respondents with a negative AF result and all with a novel pathogenic AF result understood the implications of their results (Table 1). Five of those with novel pathogenic AF results clearly detailed an appropriate follow-up plan for screening or treatment of themselves and relevant family members, while the remaining respondent indicated they were aware of the pathogenic AF result and that no action was required.

**Table 1:** Patient participant recall and understanding of opportunistic genomic sequencing results.

	Additional findings negative		Novel pathogenic additional finding	
	Rate	Illustrative quote	Rate	Illustrative quote
Recollection of test outcome				
Correctly recalled test outcome (S2)	100% (*n* = 41)		100% (*n* = 6)	
Perceived health risk				
Same or less than average	85% (*n* = 35)	AF167: I don’t think there is any impact on my future health	17% (*n* = 1)^a^	
Above average	2% (*n* = 1)	AF145: I have so many health problems now and my memory is not great and slowly getting worse.	83% (*n* = 5)	
Unsure	12% (*n* = 5)	AF164: … presuming we can now compare to the general population, my risk is the same	0%	
Lifestyle changes				
Will or have made	24% (*n* = 10)^b^		83% (*n* = 5)	AF021: I was offered an immediate (that day) option to have an echocardiogram… now under the care of regular cardiology… Father and brother have also now been tested AF032 (S2): I will follow the instructions and have a muscle byopsie done (sic), I notified my family members of the likeability (sic) of them being also susceptible. (S3): once I received the results [my child] was referred to the children’s hospital and we found out that he also carries the gene. AF057: I need to have regular blood tests to ensure that iron levels are not elevated
Not required	76% (*n* = 31)	AF140: I am continuing to live my lifestyle as normal as I can	17% (*n* = 1)^c^	AF117: No need as long as I’m aware of the condition

Three respondents with known or heterozygous HFE results have been excluded from this table.

^a^This patient was advised the RET variant was low penetrance and unlikely to result in increased risk. They were linked in to appropriate follow-up with their GP for screening.

^b^None of these respondents provided any detail on changes made. GC notes also provide no insights.

^c^Based on personal and family history, muscle biopsy was not deemed necessary for the patient with this RYR1 variant.

The majority of patient participants who completed Survey 2 indicated they received sufficient information and had no remaining concerns about their result (44/49 90%; 42/48 88%). There were a small number of comments from people with no pathogenic AF found (*n* = 5) suggesting a phone call did not completely meet their needs. In addition, comments from two people suggested they were still conflating diagnostic testing and OGS: ‘*current health problems were not picked up*’; ‘*I still have no answers*’.

GCs when interviewed also expressed concerns about patient understanding that OGS was distinct from diagnostic testing. Three GCs mentioned patients were sometimes aware of the distinction but also saw it as their ‘*last chance*’ to get an answer. One GC reported some patients were surprised when their results were negative, expecting answers to an existing condition, and ‘*still found a lot of people conflating the two*’ tests—diagnostic and OGS. One GC reported some patients seemed falsely reassured initially if their results were negative.

‘There were definitely some people who made a bit of an offhand comment like a woman saying, I don’t need to worry about having the testing that my daughter’s having now. We really did have to clarify, actually this doesn’t rule out things.’—GC01

### Decision recall and regret

Almost 75% of survey 3 patient respondents immediately remembered being offered OGS five or more months after their last GC contact, and this number rose to 86% after prompting. All patient respondents remembered their decision regarding reanalysis of their data. Two (one without a pathogenic AF and one with a pathogenic AF) had difficulty remembering the outcomes of their testing; the patient who had a pathogenic AF had previously reported being linked-in to appropriate management. Most survey 3 respondents (38/41, 93%) had low decision regret; three respondents had moderate-high decisional regret, one who had not opted-in to OGS and two who did not have pathogenic AF results.

### Future OGS service provision

**Fig. 3:**
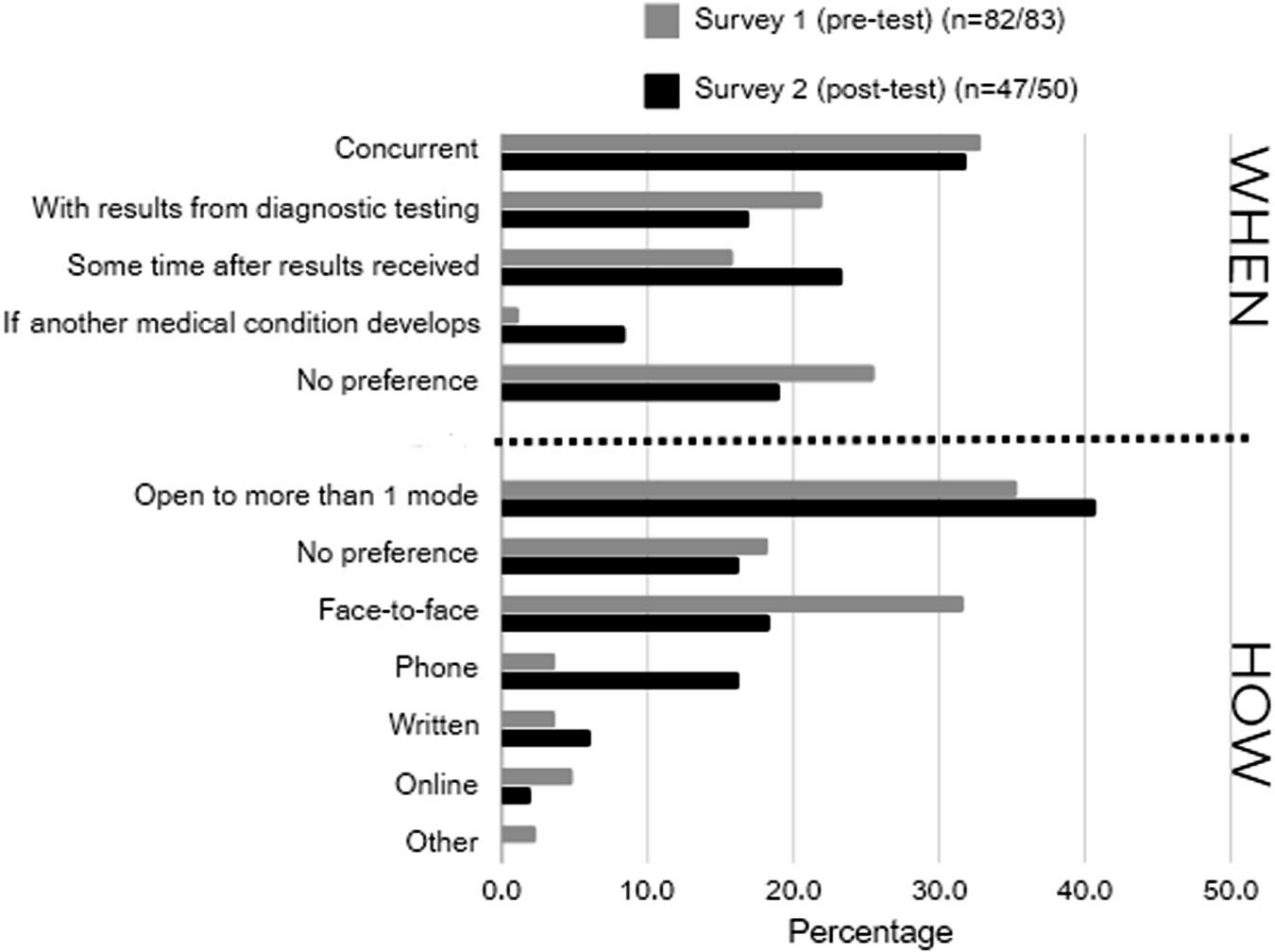
Patient participant preferences for future opportunistic genomic screening service provision.

There was no consensus on future provision of an OGS service across participating professional or patient respondents. At the survey 2 timepoint, the majority of patient respondents (86%) believed OGS should be offered in the future but there was no clear preference for when the offer should be made nor how information about OGS should be provided (Fig. 3). Of note, patient preferences changed between the survey 1 and survey 2 timepoints, with more patients preferring a delayed offer of OGS and telehealth provision at survey 2. Drawing on their experience of the pilot service, patients made a number of suggestions for future service delivery. Comments included the need for range of approaches to information delivery (Tables 2 and 3) and support needed, particularly in the context of negative diagnostic and negative OGS results (Table 2).

**Table 2:** Considerations for future service delivery based on patient participant experience.

Considerations for future service	Illustrative quotation
*Method of result delivery*	
• Written result summary to be made available as standard • Offering several options for timing and method of result delivery during consenting process to tailor to participant’s preference	AF042: Face-to-face and printed report should be given at the same time AF114: I felt the call I had was appropriate and well supportive. I appreciated not needing to go to the hospital again for the results. AF111: I would have preferred the findings to be in written form. I find it easier to read the outcomes than to listen through a phone call AF070: If negative, a simple email ‘no abnormalities found, you have option to make an appointment to discuss further’.
*Support*	
• Opt-in opportunity offered for another appointment with GC to discuss results • Systems to support those living with ongoing uncertainty including absence of a diagnosis	AF020: once I was given the results I needed time to process it then would have liked another opportunity to speak to someone. AF145: A follow-up phone call a few weeks later to make sure they have understood everything and are ok with it. AF113: I felt as though I’d been left in limbo, the test comes back positive that gives you something to work on but if the test comes back from (sic) negative it gives you nothing work on.

Note: All comments are from patients with negative AF results.

**Table 3:** Theme and example quotes representing perspectives on a future opportunistic genomic screening service.

Patient participants	Genetic counsellors	Medical scientists
*Timing of the offer of OGS—with diagnostic testing*		
AF021: Additional testing… would take time and add to people’s anxiety. Offering additional findings at the outset would in theory minimise timelines.	GC06: It’s gonna be much more cost effective if you just offer it initially with the, with the exome testing.	I think definitely together would be easier rather than having to go back and forth between reports and tracking the sample different stages
*Timing of the offer of OGS—sometime after completion of diagnostic testing*		
AF020: Time to process the findings from the test for the original condition first before contemplating other possible conditions was valuable	GC05: There’s…too much to discuss other than, you know, to be thinking about something that’s completely unrelated and completely different. So I think having it separated out by time and space and even by genetic counsellor is a good thing, so that it helps in people’s understanding that this is something different.	At the end of the day it comes down to priority of testing. Predictive [OGS] will never be higher priority than diagnostic testing, it’s just not possible, so then how do you then balance the workloads and the turn around times and things like that?
*Timing of the offer of OGS—other comments*		
AF070: If existing condition gene testing is negative then yes, offer further testing immediately. If however it is positive, let the patient get over that news first and offer further testing some time later AF096 (declined reanalysis for AF): If offered at the same time as the first testing was offered I probably would have said yes to both without thinking about it fully. Or I would have said no to both, as I didn’t want the result of the additional findings study.	GC04: I think both have their pros and cons…this [OGS] is a separate thing and I think it’s helpful, it helps make that clear to the patient that they’re separate things…but equally, you just sort of get this sense that you’re, I don’t know, sort of taking up their precious time. GC03: … to talk about it with patients at the time of their first testing and just let them know that this is an option that will be offered to them down the road. So that when it is being offered it’s not the first time they’ve heard about it and they’re clear that it is different from the other testing that they have had.	The only alternative would be to hire dedicated resources for predictive [OGS] services which may make it price prohibitive in the current environment, so having dedicated people that really just focus on predictive [OGS] which is a separate business model, you can conduct the work and actually guarantee it going on
*Information provision*		
*Flexible access to GCs*		
AF178: I’m able to make appts during business hours but realise there are many who wld … require more flexibility.	GC01: it almost might even make sense to have more of a drop in counselling service for people to have this done, because if they’re not interested, they’re not interested.	
*Digital tools*		
AF115: Email linking to an app or portal with a login for each participants	GC04: it comes down to, whether, you know, a written piece of paper as a decision aid is … realistically what people are gonna use or whether it’s some other method… like an app or something like that which they might actually have to be a bit more engaged in. GC05: if there were other alternatives for pre counselling …, that could be useful. So for example, a little video or some sort of thing that they could click on at home on their computers or you know, information sheets .., I think lots of people, … they’re interested and generally pretty informed …sort of people who are .. wanting to participate in this sort of thing.	

GC similarly did not express a clear preference for any one timepoint for offering OGS (Table 3). Comments noted offering OGS at the same time as diagnostic testing would be ‘*much more cost effective*’ and ‘*solve the problem’* of ‘*people not wanting to come in for multiple rounds of testing’*. Offering OGS after diagnostic testing was perceived as less overwhelming for patients, especially those who maybe already dealing with a health crisis. GCs further felt it would allow patients to distinguish between the two tests more clearly. GCs discussed a range of approaches to providing patients with information about OGS, although they primarily raised these as adjuncts to, rather than substitutes for counselling (Table 3).

Medical scientists were undecided about how best to provide OGS, mainly because of resource limitations. They saw the benefits of process efficiency when diagnostic and OGS analyses were conducted together, however, they were adamant that OGS analysis should not be performed at the expense of timely delivery of diagnostic testing (Table 3).

### Training needs

Medical scientists and GCs thought that additional training would be necessary ahead of implementation of OGS. Medical scientists discussed the different mindsets required for diagnostic curation versus identification and reporting of AF. Diagnostic curation includes doing ‘*your due diligence*’ to interpret variants where there are conflicting reports of pathogenicity in the context of a clinical phenotype, whereas identification and reporting of AF should be focused purely on reporting variants of known pathogenicity in people at population risk. Specific training in reporting for OGS or a separate curation workforce were suggested as solutions to this conflict. GCs indicated further training would be valuable in areas such as techniques on challenging a patient’s decision about OGS to elaborate on their values, and the depth of information to share with patients.

## Discussion

This study is the first to provide insights into a novel model of OGS service delivery, whereby diagnostic testing and OGS are treated as distinct events and offered asynchronously. Our process evaluation of this proof-of-concept OGS service explores *how* such a service might be offered in future, including stakeholder perspectives, in the context of existing service infrastructure. The results provide unique insights into several aspects of OGS service delivery that can assist decision-makers to determine which process might be best suited to their objectives and health services.

Firstly, although theoretical interest in OGS was very high (95%), actual uptake by the same patients was considerably lower (42%). This contrasts with studies offering OGS concurrent with diagnostic testing which have reported uptakes ranging from 69 to 94% [9, 16, 24]. Our 42% uptake may reflect the opportunity to more fully consider the implications of OGS. Our novel two-step study design enabled us to explore the reasons why people were NOT interested in learning about the OGS service. The reasons cited by some of those who declined suggested they made a considered decision that aligned with their values. This supports the possibility that the low rate of declining in models where OGS is offered concurrent with diagnostic testing is due to acceptance of OGS without full consideration due to convenience of the concomitant offer. Others who declined our OGS service did cite practical constraints that could theoretically be accommodated with a true two-step model with an open-ended offer, or through provision of telehealth consultations. Even so, speculatively, resolving these barriers would result in a maximum uptake of 57%.

Our results suggest separating the decision about diagnostic testing from the decision about OGS leads to high patient comprehension, illustrated by high recall of participation and understanding of results. Evaluation of concurrent delivery models has shown not all patients participate in follow-up for their pathogenic AF [16, 24]. Patients with novel pathogenic AF identified through our two-step service all reported pursuing appropriate management for their AF. Despite the provision of OGS specific face-to-face genetic counselling and decision support materials, a small group showed persistent misunderstanding of the extent of testing and confusion between diagnostic testing and OGS, although this proportion was lower than reported in studies of concurrent models [13, 25]. Further research is required to understand the reasons for such misunderstandings, as well as strategies to address them.

Patient and professional participants were open to a range of options for future OGS service delivery. In terms of the timing of the offer, this suggests OGS can be offered at whichever time best supports the local health service to meet its priorities. Concurrent offers may result in high uptake; an open-ended offer made after the decision about diagnostic testing would provide patients time to consider OGS. Our study used a resource intensive model of information provision and support which was not intended to be scalable but to offer insights for future service delivery. Most patients wanted to access information in more than one way, stressing the need for a flexible approach, which was also emphasised by participating GCs (Fig. 3, Tables 2, 3). Digital decision aids could make information about OGS accessible to patients at a time that suits them. The Genomics ADvISOR decision aid, for example, has been shown to reduce subsequent counselling time and improve knowledge of analysis for AF [26]. Chatbots are another way to provide flexible access to information about OGS and can support genetic counselling through collecting family history information relevant to the OGS offer [27]. A range of approaches for result delivery were also favoured. Of note, a reduction in preference for face-to-face contact was noted as patients experienced different modes of contact throughout the service. High acceptance of telehealth for return of results has been noted previously [28], as has the association between experience of a model of care and acceptance of it [29]. This study was conducted prior to the significant shift to telehealth caused by the COVID-19 pandemic; it is likely that acceptance of telehealth would now be higher [30]. Many patients in our study indicated they would have been happy to receive negative AF results without GC involvement. However, it is worth noting that 10% of patients without a pathogenic AF in our study wanted further guidance on results or future management. Our study contained a high proportion of people who continue on their ‘diagnostic odyssey’ [31], remaining undiagnosed after diagnostic testing. Complexity has been noted to influence patient preference for mode of service delivery (29). Complexity in our study thus relates not only to type of result (pathogenic AF, no AF) but also to the patient’s diagnostic and medical status. Specific approaches for identifying complex situations and strategies for managing these may be informed by further research and by existing service delivery models or evidence from the reproductive genetic screening setting. Offering a variety of options for information provision and return of results is likely to improve engagement, decision-making and comprehension of OGS and reserve GC availability for patients who require increased levels of assistance, maximising service viability.

Investigating the process of a two-step model for OGS allowed us to explore OGS specifically, separated from diagnostic testing, providing insights into the decision making of those who accepted and those who declined the offer of an OGS service. Though our patient cohort reflects a range of income and education levels, it was restricted to those who spoke English. Decision support materials were provided to patients, but their use and impact were not evaluated. Larger studies than ours are required to investigate outcomes such as health impacts and healthcare costs associated with OGS.

## Conclusion

The decision to provide OGS is multi-dimensional. Evidence about the outcomes of OGS is needed to make decisions about *whether* to implement OGS and outcomes are impacted by *how* OGS is offered. That is, outcomes are not independent of process. This study is the first to evaluate a novel two-step process for offering OGS, providing insights into decision-making, uptake and delivery specific to OGS.

The predominant model explored to date for OGS is to offer it concurrent with diagnostic testing. This makes sense in research and in user-pays models of healthcare. However, in socialised healthcare systems these distinct decisions do not need to be conflated. Our study demonstrates that separating them in time (enabling OGS-specific genetic counselling) results in relatively low uptake of OGS but a high recall of OGS decision-making and comprehension of results. Nonetheless, the scope of OGS was misunderstood by one third of participants; it remains to be seen if alternative, more scale-able models of information provision can improve knowledge of the OGS offer. The use of alternative models to support decision-making and return of results could also minimise some of the resource implications of separating the OGS decision in time from diagnostic testing. The two-step approach we explored does not provide a solution to all the challenges of delivering OGS; decisions about whether, when and how to offer OGS remain complex and linked to health service resourcing and priorities. Our study does provide evidence that the two-step approach warrants further exploration as an alternative to the status quo whilst highlighting outstanding OGS challenges, in particular how best to meet patient information and support needs while balancing resource requirements.

### Supplementary information

Supplementary Materials

## Data Availability

The datasets generated and/or analysed during the current study are available from the corresponding author on reasonable request.
