# Supplementary material for: Evaluation of a two-step model of opportunistic genomic screening
Source: Eur J Hum Genet. 2024 Mar 25;32(6):656–64. doi: 10.1038/s41431-024-01592-0 (PMC11153562; doi:10.1038/s41431-024-01592-0)
Supplement: Supplementary file 1 — Supplementary Materials [file 41431_2024_1592_MOESM1_ESM.docx]

**SUPPLEMENTARY FILES**

**Supplementary File 1**

Decision support material provided to potential patient participants who opted to receive further information about receiving additional after being approached about the study.

Receiving Additional Findings

Decision support information

**Thank you for your interest in the Additional Findings sub-study**

As part of the ‘Integrating Genomics into Clinical Care’ research project, you had genomic testing in relation to a specific medical condition and your genomic data was stored.

Your participation in the Additional Findings sub-study will help us understand whether it is useful to go back to stored data to look for information about conditions that may affect you in the future. This is called reanalysis of genomic data for ‘additional findings’. This will be described to you by your doctor or genetic counsellor and you will have the option of receiving additional findings.

It is important to understand that you are not being offered additional findings because we believe you are at an increased risk of other genetic conditions.

We are offering you additional findings because people across the world have shown interest in having their genomic data reanalysed for information unrelated to the original reason for testing, and your genomic data is stored and available to look at again.

This information is designed to help you understand the offer of additional findings, and help you to decide whether you would like to receive information about potential health problems you may develop in the future.

**Please read this document and bring it with you to your appointment.**

**What conditions will the reanalysis of my genomic data detect?**

We will look for changes in a specific list of genes that can cause diseases with a known treatment or intervention.

**For example:**

- The ***BRCA1*** and ***BRCA2*** genes will be analysed.

If you are found to have a change in either *BRCA1* or *BRCA2*, you would be at increased risk of developing certain cancers, such as breast cancer and ovarian cancer.

- ***Genes that can cause genetic heart disease*** will also be analysed. If you have a change in one of these genes you would be at increased risk of developing genetic heart disease. You would be referred to a medical specialist for further advice regarding how to manage your increased risk.

**What is the chance that a disease-causing change will be found?**

The chance of finding a disease-causing change from this reanalysis is very low. This means you are most likely to get a normal result; that is, a result that does not require further action.

**If you receive a normal result, there is still a chance that you may develop a(nother) genetic disease in the future.** This is because the test does not check for all conditions and cannot identify all changes in the genes tested.

If we do find a disease-causing genetic change, this may provide information about what to expect in the future, could help guide medical advice for you and may have implications for other family members.

**What happens if there is a finding on the test?**

While the most likely outcome is a normal result, if something is found, we will refer you to the appropriate specialists outside of this study to offer medical follow-up information and support.

We would also link you with services for genetic counselling, testing and support for other family members, where appropriate.

**Will this affect my ability to get insurance?**

Any genetic test results must be disclosed when applying for life, disability, trauma or income protection insurance. Companies can use this information to decide whether to offer you insurance, to set your premiums, or to exclude certain conditions from your policy.

Your results will not affect private health insurance. Any existing policies you have will not be affected.

Detailed information about the impact of your genomic sequencing results on insurance can be found at the Centre for Genetics Education at:
<http://www.genetics.edu.au/Publications-and-Resources/Genetics-Fact-Sheets/FactSheetInsurance>

**What are the advantages and disadvantages of having my genome reanalysed for additional findings?**

| **Advantages** | **Disadvantages** |
| --- | --- |
| Forewarning about potential to develop a serious medical condition  Knowledge about future disease risk ***may*** allow:   1. Earlier detection (e.g. through screening) 2. Prevention of a potential medical condition in the future 3. Earlier treatment if needed, which can be more beneficial than starting treatment at a later stage   The information may be useful for your family | It is not possible to detect all health problems  If you are found to have a genetic change:   1. The condition may never develop in the future 2. You may experience anxiety about the health problem prior to onset 3. You may require more assessments and/or further testing to confirm or exclude a diagnosis 4. You may have testing or treatment prior to the medical condition developing, even if you do not appear to be experiencing signs or symptoms of this condition 5. Your extended family could also be at risk of the condition 6. Your result could have an impact on other parts of your life (e.g. ability to obtain life insurance, choice of career) |

Other practical considerations

**Do I need to have another blood test to take part?**

No, we will use your stored genomic data, that you provided when you were recruited to the ‘Integrating Genomics into Clinical Care’ project.

Your stored data can be looked at many times. However, you may be required to provide a saliva sample to the laboratory for their quality control measures.

If a genetic change is found, another blood sample may also be needed to confirm or exclude a diagnosis. In this situation, you may also be recommended to have other medical procedures, depending on the gene change found.

**Is there a cost involved?**

There is no cost involved to have your stored genomic data looked at through this sub-study.

**How will I receive the results?**

We will organise an appointment to provide your results regardless of what we find.

**Do I want to receive information about conditions that may affect me in the future?**

This page is designed to help you think about your own views on additional findings and what information is right for you. You may find it helpful to complete it before your appointment.

People often find decision making easier when they have considered their preferences before a discussion with the genetics team.

Tick the box for statements that are true for you:

| **Column A** | **Column B** |
| --- | --- |
| I would like to have information about conditions that may occur in the future if there is a clear pathway for treatment or intervention | I am not interested in having genetic information about conditions that may occur in the future |
| I would prefer to know about my future health | Getting information about health problems that may occur in the future would make me very anxious |
| I would prefer to know about a health problem that may occur in the future so I can plan for it | I would prefer to address health problems as they arise, not beforehand |
| I would like to have information about genetic conditions that might be useful to my family | I think having information about a future health problem will have a negative impact on my family |

If you have more ticks in column A you are leaning toward finding out about conditions that may occur in the future.

If you have more ticks in column B you are leaning toward not having your genomic data reanalysed.

If you have a similar number of ticks in each column, this can be discussed at your appointment.

**Where can I get more information?**

You can discuss reanalysis of your genomic data with a genetic counsellor or doctor at your next appointment, or by contacting:

Please bring this booklet to your appointment.

[Name]

Associate Genetic Counsellor

Ph: XX XXXX XXXX

**You do not have to receive additional findings if you come in for an appointment**

**Supplementary File 2:**

Gene list and associated conditions for Additional Findings analysis provided to patients

# Gene List for Additional Findings

**It is important to remember that this test cannot identify all changes in the genes tested. If you have a personal or family history of one of the conditions on this list, the genetic counsellor or doctor that you see may recommend that you have different testing. A normal result does not mean that you cannot develop any of these diseases.**

**The laboratory report may not include all variants identified through this test; only changes (variants) that are highly likely or known to cause the disease will be included in the report.**

**Inherited Cancer Genes**

We will look for changes (variants) in 23 genes linked to an increased risk of certain types of cancer. Having a disease-causing variant found in one of these genes in most cases does not mean a person will definitely develop a cancer, but that the risk is higher for that person than for someone in the general population. If you are found to have a disease-causing change in one of these genes, doctors may recommend regular check-ups for you and also discuss the option of prevention strategies such as the use of medication and/or surgery.

| **Condition** | **Genes** |
| --- | --- |
| Hereditary breast and ovarian cancer  *(cancers of the breast, ovary, prostate and other organs)* | *BRCA1, BRCA2* |
| Li-Fraumeni syndrome  *(cancers of the breast, bones, soft tissue such as muscle, brain and other organs)* | *TP53* |
| Peutz-Jeghers syndrome.  (*growths in the gastrointestinal tract; cancers of the gastrointestinal tract, pancreas, breast, cervix, ovary and other organs)* | *STK11* |
| Lynch syndrome  *(cancers of the colon, rectum, stomach, uterus, ovary and other organs)* | *MLH1, MSH2, MSH6, PMS2* |
| Familial adenomatous polyposis  *(growths in the colon; cancers of the colon, rectum and other organs)* | *APC* |
| MYH -associated polyposis  *(growths in the colon; cancers of the colon, rectum and other organs)* | *MUTYH (For this condition, people need variants in 2 copies of the gene to be affected). Therefore, people with a variant in only one copy of the gene will not be reported.* |
| Juvenile polyposis  *(growths in the gastrointestinal tract, cancers of the colon, rectum and other organs)* | *BMPR1A, SMAD4* |
| Von Hippel–Lindau syndrome  *(haemangioblastomas which are growths made of newly formed blood vessels in can develop in the brain, spinal cord, eyes and other organs; growths in kidneys, pancreas and other organs)* | *VHL* |
| Multiple endocrine neoplasia type 1  *(growths/cancers in parathyroid glands, pituitary glands, pancreas and other organs)* | *MEN1* |
| Multiple endocrine neoplasia type 2, Familial medullary thyroid carcinoma  *(growths in adrenal glands; cancer of the medullary thyroid and other organs)* | *RET* |
| PTEN hamartoma tumour syndrome  *(cancers of the breast, thyroid, uterine lining, kidney and other organs)* | *PTEN* |
| Hereditary paraganglioma- pheochromocytoma syndrome  *(growths called paragangliomas in the head, neck, and other organs; cancers of the kidney, thyroid and other organs)* | *SDHD, SDHAF2, SDHC, SDHB* |
| Tuberous sclerosis complex  *(growths in the skin, brain, kidney and other organs)* | *TSC1, TSC2* |
| Neurofibromatosis, type 2  *(growths in brain, eyes, skin and other organs)* | *NF2* |

**Inherited Cardiac Genes**

Having a disease-causing variant found in one of these genes does not mean a person will definitely develop heart disease, but that the risk is higher for that person than for someone in the general population. If you were found to have a disease-casing change in one of these genes, doctors may recommend that you have your heart checked regularly and you may need to take certain medications, make lifestyle changes and in some cases undergo surgery.

**Cardiomyopathy**

We will look for changes (variants) in 16 genes that could cause disease of the heart muscle (cardiomyopathy). Cardiomyopathy means that your heart can not pump enough blood around the body.

| **Condition** | **Genes** |
| --- | --- |
| Hypertrophic cardiomyopathy, dilated  cardiomyopathy | *MYBPC3, MYH7, TNNT2, TNNI3, TPM1, MYL3, MYL2, ACTC1, PRKAG2, GLA, LMNA* |
| Arrhythmogenic right ventricular  cardiomyopathy | *TMEM43, DSP, PKP2, DSG2, DSC2* |

**Arrhythmia syndromes**

We will look for changes in 4 genes that could cause abnormalities in the heart’s electrical activity that could cause a fast or irregular heart rate.

| **Condition** | **Genes** |
| --- | --- |
| Catecholaminergic polymorphic ventricular  tachycardia | *RYR2* |
| *Romano-Ward long-QT syndrome types 1, 2, and 3, Brugada syndrome* | *KCNQ1, KCNH2, SCN5A* |

**Hypercholesterolemia**

We will look at changes (variants) in 3 genes that could lead to increased cholesterol in the blood. Cholesterol is naturally found in the walls of cells, but if builds up in the arteries it can lead to serious medical conditions such as coronary heart disease.

| **Condition** | **Genes** |
| --- | --- |
| Familial hypercholesterolemia | *APOB, LDLR, PCSK9* |

**Connective tissue disorders**

We will look at changes in 7 genes that could cause connective tissue disorders. These disorders affect connective tissues, which are structural portions of our body, that essentially hold the cells of the body together, such as muscles, fat, bone and cartilage. If you were found to have a disease-causing change in one of these genes doctors may recommend that you have regular check-ups to allow for early diagnosis and treatment.

| **Condition** | **Genes** |
| --- | --- |
| Ehlers-Danlos syndrome, vascular type | *COL3A1* |
| Marfan syndrome | *FBN1, TGFBR1* |
| Loeys-Dietz syndrome | *TGFBR1, TGFBR2, SMAD3* |
| Familial thoracic aortic aneurysm | *ACTA2, MYH11* |

**Endocrine disease**

We will look for changes in 2 genes that can cause endocrine disorders. The first disorder is Wilson disease, which involves a build-up of copper in the body, particularly the liver, brains and eyes. The second is Ornithine Transcarbamylase Deficiency which causes a build-up of ammonia in the blood. Both can lead to severe medical conditions such as liver disease. If you were found to have a disease-causing change in one of these genes,doctors might recommend that you take certain medication and have regular check-ups.

| **Condition** | **Genes** |
| --- | --- |
| Wilson disease | *ATP7B* |
| Ornithine transcarbamylase deficiency | *OTC* |

**Malignant Hyperthermia**

We will look for changes in 2 genes that could cause a severe reaction to particular anaesthetic drugs typically used during surgery. If you were found to have a disease-causing variant in one of these genes, you would be recommended to avoid certain medications when having an anaesthetic.

| **Condition** | **Genes** |
| --- | --- |
| Malignant hypothermia susceptibility | *RYR1, CACNA1S* |

**Haemochromatosis**

We will look for particular variants in one gene that causes Hemochromatosis. People with hemochromatosis absorb too much iron form their diet. Over time, an overload of iron in the body can lead to organ and tissue damage. If you are found to have hemochromatosis, your doctor may advise you to have regular blood tests and to become a regular blood donor.

| **Condition** | **Genes** |
| --- | --- |
| Haemochromatosis | *HFE*  *(For this condition, people need variants in 2 copies of the gene to be affected). We will check whether there are 2 copies of the C282Y variant and one copy of the C282Y variant plus another different variant.* |

**SUPPLEMATRAY FILE 3**

Demographics of those approached and offered reanalysis for AF in the proof-of-concept OGS service. Those who opted-in agreed to learn more about OGS – they still had the opportunity to decline analysis for AF.

| **Table 1 Patient Demographics** | | | |
| --- | --- | --- | --- |
|  | Opted-in (n=83) | Opted-out or lost to follow up (n=117) | Pearson's Chi Squared Test p value |
|  | Freq (%) | Freq (%) |  |
| **Age in years – Mean (range)** | 50 (20 – 89) | 47 (19 – 83) | 0.17 |
| **Sex** | | | |
| Male | 37 (45%) | 63 (54%) | 0.2 |
| Female | 46 (55%) | 54 (46%) |  |
| **Location** | | | |
| Metropolitan Melbourne | 67 (81%) | 87 (74%) | 0.15 |
| Regional Victoria | 16 (19%) | 25 (21%) |  |
| Interstate | 0 (0%) | 5 (4%) |  |
| **Aboriginal and/or Torres Strait Islander identity** | | | |
| Aboriginal and/or Torres Strait Islander | 1 (1%) | 1 (1%) | - |
| Neither Aboriginal nor Torres Strait Islander | 82 (99%) | 111 (95%) |  |
| Not stated/inadequately described | 0 (0%) | 5 (4%) |  |
| **Country of birth** | | | |
| Born in Australia | 77 (93%) | 86 (74%) | 0.1 |
| Born overseas | 6 (7%) | 16 (14%) |  |
| Not stated/inadequately described | 0 (0%) | 15 (13%) |  |
| **Highest level of education** | | | |
| Below secondary | 20 (24%) | 16 (14%) | 0.16 |
| Secondary equivalent | 15 (18%) | 26 (22%) |  |
| Post-secondary | 29 (35%) | 20 (17%) |  |
| Postgraduate degree | 19 (23%) | 16 (14%) |  |
| Missing | 0 (0%) | 39 (33%) |  |
| **Income**  **– state quintiles** | | | |
| 0-20% (< AU$36,000) | 11 (13%) | Not collected | - |
| 21-40% (AU$36,000 - AU$63,000) | 8 (10%) |  |  |
| 41-60% (AU$63,000 - AU$100,000) | 15 (18%) |  |  |
| 60-80% (AU$100,000 - AU$154,000) | 5 (6%) |  |  |
| 80-100% (> AU$154,000) | 16 (19%) |  |  |
| Prefer not to say | 27 (33%) |  |  |
| **Received diagnosis from initial genomic diagnostic testing?** | | | |
| Yes | 10 (12%) | 23 (20%) | 0.15 |
| No | 73 (88%) | 94 (80%) |  |
| **Time from completing initial testing to being approached for AFs in months – median (IQR)** | 5.2 (2.4 – 13.2) | 6.2 (2.5 – 16.0) | 0.94 (ranksum) |
| Missing time | 3 | 11 |  |

**Supplementary File 4**

| Participant | Gene variant | Associated condition | Known (when) / Novel | Outcome |
| --- | --- | --- | --- | --- |
| AF007 | *RET* c.5410G>A p.(Val804Met | Multiple endocrine neoplasia type 2 | Novel | Referred to GP to review thyroid and calcitonins annually. Children and siblings may be referred for predictive testing to genetics service |
| AF021 | *MYH7* c.2539A>G p.(Lys847Glu | Hypertrophic cardiomyopathy | Novel | Seen by cardiologist on day of results disclosure. Had undergone ECHO which showed early signs of hypertrophic cardiomyopathy. Patient arranged ongoing care with caridiologist, referred to traceback (mother had ovarian cancer) and discussed surveillance of relatives. |
| AF032 | *RYR1* c.10042C>T p.(Arg3348Cys | Malignant hypothermia | Novel | Had muscle biopsy. Referred to Malignant Hypothermia clinic  Also had son tested, who was found to carry the same gene variant. |
| AF117 | RYR1 c.10042C>T p.(Arg3348Cys) | Malignant hypothermia | Novel | Based on personal and family history, muscle biopsy was not necessary. Referred (including family members) to Malignant Hypothermia specialist clinic |
| AF131 | *HFE* c.845G>A p.(Cys282Tyr) | Haemochromatosis | Novel | Referral for iron levels to be checked and management through General Practitioner |
| AF163 | *HFE* c.845G>A p.(Cys282Tyr) | Haemochromatosis | Novel | Referral for iron levels to be checked and management through General Practitioner |

Table providing details of the 6 novel AF results reported from the 81 analyses undertaken
